# Design of Three-shell Icosahedral Matryoshka Clusters A@B_12_@A_20_ (A = Sn, Pb; B = Mg, Zn, Cd, Mn)

**DOI:** 10.1038/srep06915

**Published:** 2014-11-07

**Authors:** Xiaoming Huang, Jijun Zhao, Yan Su, Zhongfang Chen, R. Bruce King

**Affiliations:** 1 Key Laboratory of Materials Modification by Laser, Ion and Electron Beams (Dalian University of Technology), Ministry of Education, Dalian 116024, China; 2 Beijing Computational Science Research Center, Beijing 100089, China; 3 Department of Chemistry, Institute for Functional Nanomaterials, University of Puerto Rico, San Juan, PR 00923, USA; 4 Department of Chemistry and Center for Computational Chemistry, University of Georgia, Athens, Georgia, USA

## Abstract

We propose a series of icosahedral matryoshka clusters of A@B_12_@A_20_ (A = Sn, Pb; B = Mg, Zn, Cd), which possess large HOMO-LUMO gaps (1.29 to 1.54 eV) and low formation energies (0.06 to 0.21 eV/atom). A global minimum search using a genetic algorithm and density functional theory calculations confirms that such onion-like three-shell structures are the ground states for these A_21_B_12_ binary clusters. All of these icosahedral matryoshka clusters, including two previously found ones, i.e., [As@Ni_12_@As_20_]^3−^ and [Sn@Cu_12_@Sn_20_]^12−^, follow the 108-electron rule, which originates from the high *I_h_* symmetry and consequently the splitting of superatom orbitals of high angular momentum. More interestingly, two magnetic matryoshka clusters, i.e., Sn@Mn_12_@Sn_20_ and Pb@Mn_12_@Pb_20_, are designed, which combine a large magnetic moment of 28 µ_B_, a moderate HOMO-LUMO gap, and weak inter-cluster interaction energy, making them ideal building blocks in novel magnetic materials and devices.

The first evidence for the formation of bare anionic clusters of the post-transition elements, particularly those of groups 14 and 15, was obtained by Zintl and co-workers^1^,^2^,^3^,^4^ in the 1930s from the potentiometric titrations of post-transition elements with alkali metals in liquid ammonia. However, definitive structure determinations of these clusters became possible only in the 1970s, when Kummer and Diehl reported the first structurally-authenticated Zintl anion cluster of Na_4_Sn_9_^5^. The original post-transition element clusters were empty, i. e., they contained no interstitial atoms in the center of the cluster polyhedron. However, subsequent synthetic studies led to the discovery of clusters containing interstitial transition metals. Examples of structurally characterized such clusters based on 10-vertex outer polyhedra include the anionic indium clusters M@In_10_^10−^ (M = Ni, Pd, Pt)^6^ in the intermetallics K_10_In_10_M, the anionic lead clusters M@Pb_10_^2−^ in [K(2,2,2-crypt)]_2_[M@Pb_10_] (M = Ni, Pd, Pt)^7^, Fe@Sn_10_^3−^ in [K(2,2,2-crypt)]_3_[Fe@Sn_10_]^8^, the cationic pentagonal antiprismatic bismuth cluster Pd@Bi_10_^4+^ in Bi_14_PdBr_16_ ( = [Pd@Bi_10_][BiBr_4_]_4_)^9^, and the centered pentagonal prismatic clusters M@Ge_10_^3−^ (M = Co^10^, Fe^11^). Interstitial metal atoms are also found in the icosahedral M@Pb_12_^2−^ clusters (M = Ni, Pd, Pt)^7^,^12^. Furthermore, Al^3+^ has been encapsulated in Pb_12_^2−^, Pb_10_^2−^ and Sn_10_^2−^ clusters, which have been characterized by mass spectroscopy and density functional theory (DFT) calculations^13^,^14^.

Attempts to expand the knowledge of transition metal derivatives of post-transition element clusters led to the discovery of even more complicated structures. Of particular interest is the cluster [As@Ni_12_@As_20_]^3−^, isolated as its *n*-Bu_4_P^+^ salt and structurally characterized by X-ray diffraction^15^. This cluster anion has a perfect icosahedral (*I_h_*) symmetrical three-shell matryoshka doll structure consisting of an outer As_20_ regular dodecahedron encapsulating an Ni_12_ icosahedron, which in turn encapsulates an As^3−^ trianion (Figure 1). Beyond the filled d^10^ shells of the arsenic and nickel atoms, this system has a total of 108 valence electrons, among which 40 skeletal electrons^16^ correspond to a “magic” number of a jellium sphere^17^ with 1S^2^1P^6^1D^10^2S^2^1F^14^2P^6^ molecular orbital configuration.

Initially the icosahedral matryoshka [As@Ni_12_@As_20_]^3−^ structure with 108 valence electrons was considered as an anomaly, unique with an unusual combination of specific electronic and steric properties of its arsenic and nickel components. However, in 2011 Stegmaier and Fässler^18^ reported a completely analogous [Sn@Cu_12_@Sn_20_]^12−^ icosahedral matryoshka doll anion in the M_12_Cu_12_Sn_21_ intermetallics (M = Na, K). This [Sn@Cu_12_@Sn_20_]^12−^ anion is exactly isoelectronic with the [As@Ni_12_@As_20_]^3−^ anion with 108 valence electrons beyond the filled d^10^ shells of the tin and copper components. Similar bonding models appear to be applicable to both the [As@Ni_12_@As_20_]^3−^ and [Sn@Cu_12_@Sn_20_]^12−^ systems. Furthermore, the observation of the icosahedral matryoshka doll structure in both the soluble [As@Ni_12_@As_20_]^3−^ anion and the [Sn@Cu_12_@Sn_20_]^12−^ anion in the A_12_Cu_12_Sn_21_ intermetallics suggests that such structures are particularly favorable in clusters where 108 total valence electrons are available from the elements composing the three layers of the matryoshka structure.

Parallel to the condensed phased clusters^19^ such as those discussed above, gas-phase clusters have been intensively studied during the past three decades since they exhibit many fascinating physical and chemical properties that depend on their size, geometry, and composition^20^. Interestingly, some specific clusters with appreciable stability mimic the chemical behavior of elemental atoms in the periodic table and thus can be regarded as “superatoms”^21^,^22^,^23^,^24^. In these superatom clusters, the electronic states are delocalized over the entire cluster with spatial shapes resembling the atomic orbitals. The corresponding energy eigenvalues can be grouped into atomic-like shells (1S, 1P, 1D, 2S, 1F, 2P, …), depending on the degeneracy and spatial symmetry of the molecular orbitals. Unlike the atoms, the physical and chemical properties of superclusters can be tailored through selection of size and composition, making them very promising building blocks for new materials^25^,^26^.

The concept of “superatoms” was originally proposed by Khanna and Jena in their pioneering paper^21^. For example, an Al_13_^−^ cluster with filled 1S^2^, 1P^6^, 1D^10^, 2S^2^, 1F^14^, and 2P^6^ shells exhibits strong resistance to oxidation^27^. Meanwhile, with an electron affinity (EA = 3.57 eV)^22^ only slightly lower than that of the Cl atom (3.62 eV), a neutral Al_13_ cluster was considered as a superhalogen by Bergeron and co-workers^27^, though this nomenclature is not consistent with the original definition of superhallogen by Bolydyrev and Gutsev^28^, which defined superhalogen as a cluster/molecule whose electron affinity is larger than that of any halogen atom. Moreover, a neutral Al_14_ cluster behaves like an alkaline earth atom^29^.

Usually, a superatom cluster is stable because it has filled electron shells with paired electrons, corresponding to a nonmagnetic state. The concept of magnetic superatom was initially introduced by Kumar and Kawazoe^30^, who predicted Mn@X_12_ clusters (X = Ge, Sn) as icosahedral magnetic superatoms with a high magnetic moment of 5 μ_B_ and a large highest occupied molecular orbital-lowest unoccupied molecular orbital (HOMO-LUMO) gap of ~1.1 eV. By appropriately combining the localized d states with magnetism and the delocalized superatom orbitals that stabilize the entire cluster, Khanna and co-workers recently designed a series of magnetic superatoms^31^, such as VCs_8_^32^, VNa_8_^−^33, Mg_8_Fe^34^, Ca_8_Fe^35^, MnCa_9_^36^, MnSr_9_^37^, ScK_12_ and ScCs_12_^38^. All of them possess enhanced stabilities, substantial HOMO-LUMO gaps (usually about 0.4 ~ 0.7 eV), and large magnetic moments on the transition metal atom (up to 5 μ_B_).

Stimulated by the above-mentioned progresses, here we propose a series of icosahedral matryoshka clusters, i.e., A@B_12_@A_20_ (A = Sn, Pb; B = Mg, Zn, Cd, Mn). Analogous to [As@Ni_12_@As_20_]^3−^ and [Sn@Cu_12_@Sn_20_]^12−^, all of these clusters show high stability and alignment of superatom orbitals. In particular, the Sn@Mn_12_@Sn_20_ and Pb@Mn_12_@Pb_20_ clusters possess a giant magnetic moment of 28 μ_B_, which can be retained in cluster assemblies. Our results thus extend the scope of icosahedral matryoshka clusters and provide novel building blocks for cluster-based materials and devices exhibiting fascinating novel magnetic and optical properties.

## Results and Discussion

The binding energies, formation energies, HOMO-LUMO gaps, and optical absorption gaps for icosahedral matryoshka A@B_12_@A_20_ clusters (A = Sn, Pb; B = Mg, Zn, Cd) are summarized in Table 1. For a given cluster of A_x_B_y_, its formation energy is defined by: E_f_ (A_x_B_y_) = E(A_x_B_y_) – xE(A) – yE(B), where E(A_x_B_y_) is the energy of A_x_B_y_ cluster, E(A) or E(B) is the energy per atom of the constituent element A or B in the standard states, x or y is the number of A or B atoms in the cluster. Detailed information on geometry parameters and on-site charges for these clusters are given in Table S1 of Supplementary Information.

All the clusters explored here show reasonably low formation energy (typically between 0.1 and 0.2 eV/atom), indicating that they are easy to form thermodynamically. For comparison, we consider several 38-atom elementary metal clusters with a fcc-like truncated octahedron structure^39^. Higher formation energies are obtained, i.e., 0.225 eV/atom for Pb_38_, 0.281 eV/atom for Sn_38_, 0.576 eV/atom for Mg_38_, 0.497 eV/atom for Zn_38_, and 0.385 eV/atom for Cd_38_ clusters, respectively. Using global optimization combined with DFT calculations, Ferrando's group^40^ has recently achieved a series of three-shell high-symmetry matryoshka clusters, including a Ni_13_@Mg_20_ that is isostructural to the clusters considered here. However, the formation energy of this Ni_13_@Mg_20_ cluster is as high as 0.468 eV/atom from our DFT calculation. The more favorable formation energies of the present icosahedral matryoshka superatoms clearly demonstrate the stabilization effect of electronic structure, which will be discussed in detail later.

Note that the lengths of Pb-Pb (3.317 to 3.578 Å) and Sn-Sn (3.176 to 3.463 Å) bonds listed in Table S1 are longer than the equilibrium bond lengths of the hollow Pb_20_ (3.073 Å) and Sn_20_ (2.891 Å) cages from our DFT optimization. Therefore, the enlarged Pb_20_ and Sn_20_ cages are stabilized by the encapsulated C@B_12_ icosahedron (C = Sn, Pb; B = Mg, Zn, Cd), as previously found^41^ for [As@Ni_12_@As_20_]^3−^. It is also interesting to compare the structural parameters of Sn@Zn_12_@Sn_20_ with its isoelectronic counterpart, i.e., [Sn@Cu_12_@Sn_20_]^12−^. The theoretical Sn-Sn bond lengths in Sn@Zn_12_@Sn_20_ of 3.278 Å are comparable to that of 3.290 Å for [Sn@Cu_12_@Sn_20_]^12−^ computed with the same level of theory. Note that the experimental Sn-Sn bond lengths of [Sn@Cu_12_@Sn_20_]^12−^ in the condensed phase obtained by X-ray crystallography range between 3.076 to 3.133 Å^18^.

According to the on-site population analysis (see Supplementary Table S1), the B atoms in the intermediate shell donate some charge to the A atoms in the exterior shell, while the central A atom gains more charge, ranging from 0.245 to 1.362 electrons (by Mulliken definition). The trend of charge transfer remains the same for different systems, suggesting that all these clusters share the same pattern of chemical bonding. The direction of charge transfer can be easily understood by the electronegativity differences, that is, the Pauling electronegativities of Pb (2.33) and Sn (1.96) are higher than those of Mg (1.31), Zn (1.65), Cd (1.69), and Mn (1.55).

As shown in Table 1, the HOMO-LUMO gaps of the A_21_B_12_ clusters with B = Mg, Zn, Cd range from 1.27 to 1.54 eV, comparable to those of [As@Ni_12_@As_20_]^3−^ (1.44 eV) and [Sn@Cu_12_@Sn_20_]^12−^ (1.39 eV) using the same computational scheme. Previous DFT calculations yielded similar band gaps for [As@Ni_12_@As_20_]^3−^ (1.44 eV^41^) and [Sn@Cu_12_@Sn_20_]^12−^ (1.34 eV^18^). The high symmetries of these clusters brings about multiple degeneracy of the frontier molecular orbitals and severe selection rules in optical transitions. Our TD-DFT calculations further reveal that the lowest singlet excitation (HOMO-to-LUMO) is optically forbidden due to the LUMO and HOMO symmetries. The first optically-allowed excitation (i.e., optical absorption gap E_og_) and the associated energy levels are summarized in Table 1. The computed E_og_ by TD-DFT ranges from 1.72 to 1.94 eV (i.e., the visible light region), making them potentially useful as nanoscale building blocks for optoelectronic devices^25^,^41^.

As aforementioned, the experimentally found icosahedral matryoshka clusters, i.e., [As@Ni_12_@As_20_]^3−^ and [Sn@Cu_12_@Sn_20_]^12−^, are exactly isoelectronic with 108 valence electrons beyond the filled d^10^ shells (for As, Ni, Sn, Cu). The same 108-electron rule applies to all binary A@B_12_@A_20_ clusters considered here, noting that Mg has no d electrons. However, 108 does not explicitly belong to any existing magic numbers for various models, e.g., the shell model within the jellium approximation^23^, the Wade-Mingos rules^42^, the spherical aromatic model^43^, the octet or eighteen-electron rule^23^. Therefore, we explore the origin of the “magic number” of 108 for these spherical multi-shell clusters as a guide to design other matryoshka clusters exhibiting high stabilities and sizeable electronic gaps.

For simplicity, we start with Sn@Mg_12_@Sn_20_ that does not involve any d electrons in the valence shells of the component atoms. The energy levels and spatial distribution for the Kohn-Sham molecular orbitals of Sn@Mg_12_@Sn_20_ are presented in Figure 2 and Figure 3, respectively. According to the energetic sequence and the nodal shape of the orbitals, we can identify a series of superatom orbitals in the sequence 1S, 1P, 2S, 1D, 1F, 1G, 2P, 3S, 2D, 1H, 2F, 3P, 1I corresponding to the 54 lowest-lying valence molecular orbitals (108 valence electrons) up to and including HOMO. Among them, 1S, 1P, 2S, 1D, 1F, 1G, 2P, 3S, 2D, 3P orbitals are completely filled. In the icosahedral ligand field^44^, the 2F, 1H and 1I superatom orbitals are split into two, three and four components, respectively. The lowest 1I orbital (g_g_) is occupied as the HOMO of superatom. The lowest two (t_2u_ and h_u_) of 1H orbitals are occupied, while the triply degenerate middle 1H t_1u_ orbital is the LUMO of superatom. The lower g_u_ component of 2F orbitials is occupied and the higher t_2u_ one is empty as LUMO+1. Even without closure of any specific superatom orbital, a large HOMO-LUMO gap of 1.383 eV separates the occupied g_g_ component of the 1I orbital and the empty t_1u_ component of the 1H orbital of Sn@Mg_12_@Sn_20_. Therefore, the “magic number” of 108 electrons of the icosahedral matryoshka clusters originates from the high *I_h_* symmetry and consequently the splitting of molecular orbitals of high angular momentum (2F, 1H and 1I)^44^. Previously, Martin and co-workers^45^ had shown that both electronic and geometric effects can lead to extraordinary stability for a cluster (i.e., a magic cluster).

Meanwhile, the suitable matching of atomic sizes of the component elements ensures the geometry stability of such unique three-layer matryoshka with high *I_h_* symmetry. As summarized in the Supplementary Table S2, the covalent radii^46^ of the constituent elements in an icosahedral matryoshka cluster have to match with each other (in the range of 1.22 ~ 1.46 Å). Indeed, we have also investigated other possible icosahedral matryoshka A@B_12_@A_20_ clusters with B = Be, Ca (and A = Sn, Pb). However, all of them exhibited several large imaginary vibrational frequencies due to mismatch of atomic radius and thus were not further discussed here.

The energy levels for the four icosahedral matryoshka clusters, including [As@Ni_12_@As_20_]^3−^, [Sn@Cu_12_@Sn_20_]^12−^, Sn@Mg_12_@Sn_20_, and Sn@Zn_12_@Sn_20_, are compared in Supplementary Figure S1. The arrangement of molecular orbitals in each species is generally similar. The differences for those clusters are related to the specific energy of each orbital as well as the relative location of the d orbitals in the Ni, Cu, or Zn derivatives. However, the d orbitals, which locate only in a narrow energy range, have no noticeable influence on the overall superatom orbitals. On the other hand, the very deep d levels for As, Sn, and Pb do not overlap with the superatom orbitals. As discussed above, the universal arrangement of molecular orbitals must originate from the unique icosahedral nesting doll structure. This demonstrates further the existence of a class of matryoshka clusters with similar geometries and electronic properties.

Analogous to Cu, Ni, Zn, Cd elements with filled d^10^ shells, we further design two new icosahedral matryoshka clusters by using Mn with a half-filled d^5^ shell, that is, Sn@Mn_12_@Sn_20_ and Pb@Mn_12_@Pb_20_. According to Hund's rule, the Mn atoms are expected to carry certain magnetic moments. Indeed, our DFT calculations show that both Sn@Mn_12_@Sn_20_ and Pb@Mn_12_@Pb_20_ possess a giant magnetic moment of 28 μ_B_. Mulliken population analysis reveals that the magnetism mainly resides on the Mn atom, i.e., 2.880 (3.013) μ_B_ on each Mn atom for Sn_21_Mn_12_ (Pb_21_Mn_12_), whereas there are certain induced antiferromagnetic moments (about −0.3 ~ 0.8 μ_B_) on the central and exterior Sn or Pb atoms (see Table 2). Even with the half-filled d^5^ shell and large spin polarization, these two magnetic superatoms exhibit moderate HOMO-LUMO gaps of 0.382 eV (Sn_21_Mn_12_) and 0.614 eV (Pb_21_Mn_12_), respectively, which are comparable to the gap magnitude (0.4 ~ 0.7 eV) for previously proposed magnetic superatoms, i.e., VNa_8_^−^33, Mg_8_Fe^34^, Ca_8_Fe^35^, MnCa_9_^36^, MnSr_9_^37^, TcMg_8_^47^, ScK_12_ and ScCs_12_^38^.

The spin-polarized density of states for Sn@Mn_12_@Sn_20_ and Pb@Mn_12_@Pb_20_ clusters are presented in Figure 4. Even with spin polarization, the alignments of superatom orbitals in Sn@Mn_12_@Sn_20_ and Pb@Mn_12_@Pb_20_ still resemble those of their non-magnetic counterparts shown in Supplementary Figure S1. This suggests that the 3d orbitals of Mn in such species are effectively non-bonding and contribute only to the overall magnetic moment of the clusters. As demonstrated by the spin density distribution in Figure 4(c) and (d), the magnetism mainly originates from the unpaired 3d electrons from the Mn atoms, while some superatom orbitals (like 2P, 2D, 3S) also exhibit certain exchange splitting between the majority and minority spin states. We also observe some hybridization between the 3d states of Mn and the superatom orbitals (2F, 1G, 1H, 1I). Previously, Khanna and co-workers^32^,^33^,^34^,^35^ suggested that the hybridization between transition metal d states and superatom states as well as the combined action of the crystal field splitting and the exchange splitting are the key factors to stabilize a magnetic superatom and to ensure a large magnetic moment. Although the size and geometry of the current clusters are entirely different from the previous ones, this design principle for magnetic superatoms remains valid.

Previously, an [Mn@Mn_12_@Au_20_]^−^ cluster, iso-structural to the current icosahedral matryoshka, was also predicted to possess a giant magnetic moment of 44 μ_B_^48^, but with a smaller HOMO-LUMO gap (0.25 eV). Using current theoretical scheme, the calculated formation energy for Mn@Mn_12_@Au_20_ is 0.795 eV/atom, about twice of Sn_21_Mn_12_. Again, this indicates that design of highly stable superatoms requires the consideration of electronic structures, i.e., closure of electronic shell or subshell. Also note that Sun et al.^49^ designed an onion-like caged clusters Fe@Au_12_@Au_42_ in which the magnetic moment on Fe atom (3 μ_B_) is enhanced over bulk value, while Wu and Jena^50^ recently proposed a series of stable ferromagnetic hollow cages with large magnetic moments, i.e., Co_12_C_6_ (14 μ_B_), Mn_12_C_6_ (38 μ_B_), and Mn_24_C_18_ (70 μ_B_).

In addition, a [Mn_12_] single molecular magnet, i.e., Mn_12_O_12_(CH_3_COO)_16_(H_2_O)_4_, which possesses a magnetic moment of 20 μ_B_^51^, has been intensively studied^52^. To some extent, the present magnetic superatom clusters of Sn_21_Mn_12_ and Pb_21_Mn_12_ can be regarded as a kind of ligand-free single molecular magnet of [Mn_12_] with slightly enhanced magnetic moment (28 μ_B_ vs. 20 μ_B_). The high symmetries, appreciable HOMO-LUMO gaps, and giant magnetic moments makes Sn_21_Mn_12_ and Pb_21_Mn_12_ clusters promising building units for novel cluster-assembled magnetic materials and devices, which have potential applications in high-density information storage^53^, molecular spintronics^54^, quantum computing^55^, and magnetic resonance imaging (MRI)^56^, etc.

In the cluster-assembled materials, clusters are either encapsulated into a zeolite matrix^57^ or deposited on a substrate^58^. It is thus crucial to examine whether every clusters in the assembly can keep their identity without coalescing into larger clusters, like the previous case of VCs_8_^32^. More importantly, the fantastic magnetic characteristics of the individual clusters must be well retained. As a simplest prototype of cluster assemblies, here we investigate the cluster-cluster interaction by considering the dimers of Sn@Mn_12_@Sn_20_ and Pb@Mn_12_@Pb_20_. The atomic structure and binding curve for [Pb@Mn_12_@Pb_20_]_2_ dimer are shown in Figure 5 as a representative. These two clusters are stacked along a 5-fold symmetry axis with parallel pentagonal faces in a staggered orientation, which is more stable than the direct orientation by 0.180 eV. Upon formation of the dimer, there is no noticeable deformation of cluster configuration. Accordingly, the interaction strength between the two spherical clusters is rather weak, as demonstrated by the large inter-cluster distances (*d* = 3.787 Å for [Sn@Mn_12_@Sn_20_]_2_ and *d* = 4.153 Å for [Pb@Mn_12_@Pb_20_]_2_, see Figure 5) and small inter-cluster binding energies (0.359 eV for [Sn@Mn_12_@Sn_20_]_2_ and 0.291 eV for [Pb@Mn_12_@Pb_20_]_2_. Such weak cluster-cluster interaction might be attributed to the moderate HOMO-LUMO gap as well as the sphere-like icosahedral geometry of the clusters. Most excitingly, the total magnetic moments of both cluster dimers are exactly twice of the individual ones, i.e., 56 μ_B_. Indeed, the on-site local moments in the cluster dimers are barely affected by the neighboring cluster (see Table 2). To consider the possible antiferromagnetic state, we initially set different spin directions (spin up vs. spin down) for the Mn atoms in each of the two clusters in [Sn@Mn_12_@Sn_20_]_2_ or [Pb@Mn_12_@Pb_20_]_2_ dimer. However, upon relaxation of spin-polarized electron wavefunction, the spin state of the cluster dimer spontaneously transforms from antiferromagnetic to ferromagnetic. Moreover, inclusion of dispersion correction for the inter-cluster interaction only moderately increases the magnitude of binding energy E_int_ but has no effect on the magnetism of the cluster dimers. These results clearly indicate that the Sn@Mn_12_@Sn_20_ or Pb@Mn_12_@Pb_20_ clusters may keep their identities and retain their superior magnetism in cluster-assembled materials and devices. This is a vital prerequisite for their future applications.

## Conclusion

A series of three-shell icosahedral matryoshka clusters related to the experimentally known [As@Ni_12_@As_20_]^3−^ and [Sn@Cu_12_@Sn_20_]^12−^ are proposed. A DFT-based global minimum search confirms that such icosahedral matryoshka structures are ground state configurations for these A_21_B_12_ (A = Sn, Pb; B = Mg, Zn, Cd) binary clusters. In addition, the high stabilities of these spherical matryoshka clusters are demonstrated by their large HOMO-LUMO gaps and low formation energies. The molecular orbitals for different icosahedral matryoshka clusters share the same pattern of superatom orbitals. The “magic number” of 108 electrons can be attributed to the high *I_h_* symmetry and consequently the splitting of superatom orbitals of high angular momentum. Such “108-electron rule” should be extendable to other icosahedral matryoshka superatoms with different charge states, which might lead to future experimental discoveries of new metal clusters in solution as well as novel solid-state intermetallic compounds. Two related magnetic icosahedral matryoshka clusters, namely Sn@Mn_12_@Sn_20_ and Pb@Mn_12_@Pb_20_, are predicted to exhibit large magnetic moments (28 μ_B_) and moderate HOMO-LUMO gaps. More impressively, after formation of cluster assemblies, these two clusters are able to keep their identities and retain their magnetic moments due to weak inter-cluster interaction. These novel binary clusters with unique icosahedral nesting doll geometry, high thermodynamic stabilities, and interesting physical properties (such as optical gaps in the visible region and giant magnetic moments), are expected to be useful building blocks in future nanoscale materials and devices.

## Methods

*Ab initio* calculations were performed using the spin-polarized density functional theory as implemented in the DMol^3^ program^59^. The core electrons were treated by an all-electron relativistic method including scalar relativistic effects. The generalized gradient approximation (GGA) with PW91 parameterization^60^ was adopted to describe the exchange-correlation interaction and the double numerical basis set including d-polarization functions (DND) were employed. Vibrational analyses were performed for each cluster to ensure that the optimized structures are the true minima on the potential energy surface. In addition, time-dependent density functional theory (TD-DFT) calculations with PW91 functional were carried out for those A@B_12_@A_20_ (A = Sn, Pb; B = Mg, Zn, Cd) clusters to obtain the optical adsorption spectra and optical gaps.

**Figure 1:**
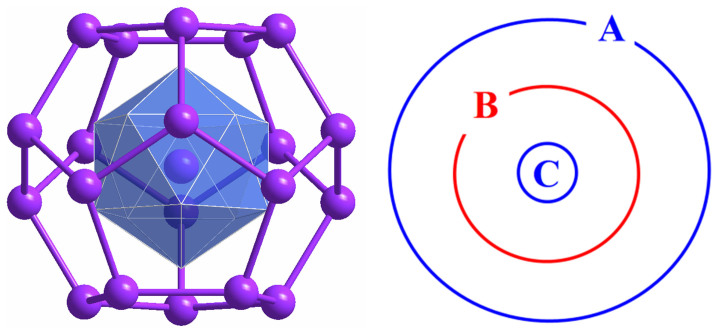
Atomic structure of an icosahedral matryoshka cluster of C@B_12_@A_20_ (left) and its schematic plot (right).

**Figure 2:**
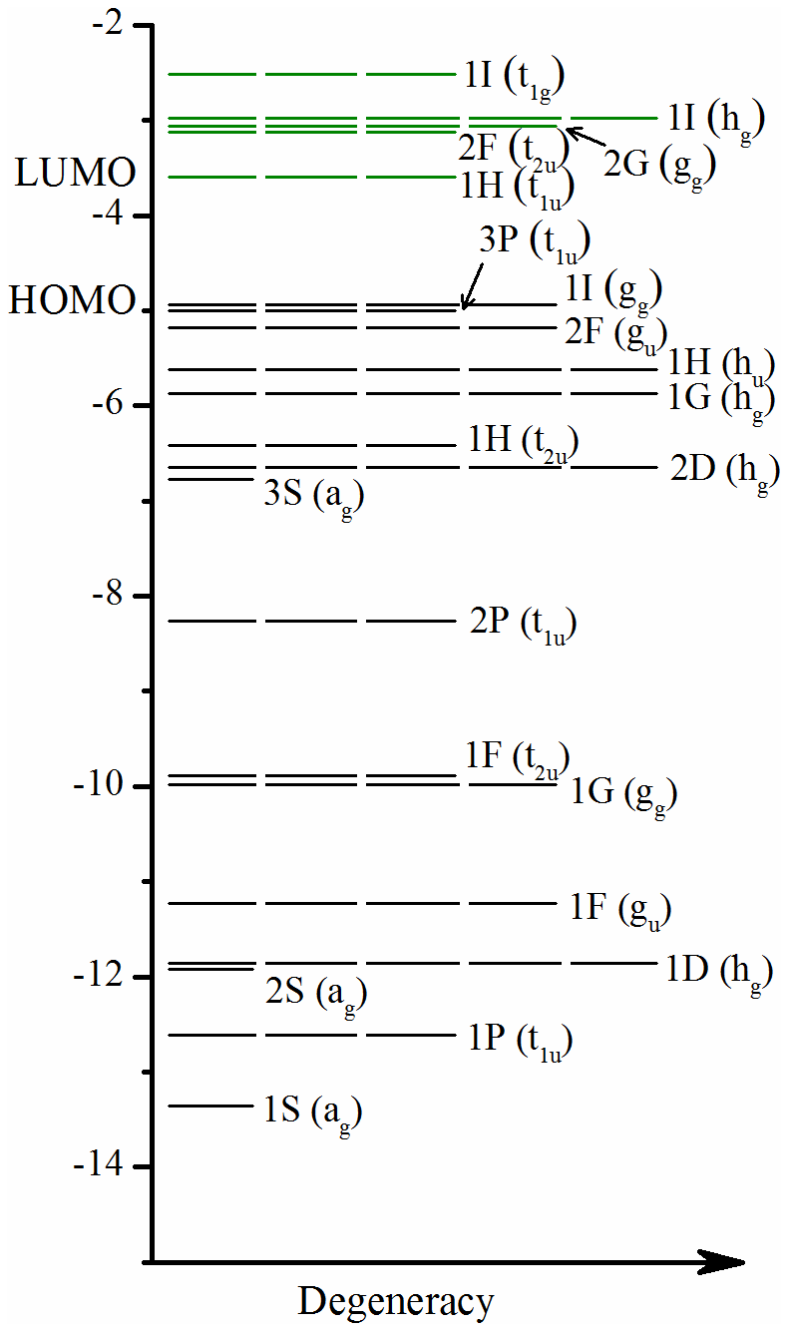
Energy levels (in eV) for the molecular orbitals of the Sn@Mg_12_@Sn_20_ cluster, which can be assigned to a series of superatom orbitals corresponding to the indicated spherical harmonics. A few unoccupied levels are shown and highlighted by green.

**Figure 3:**
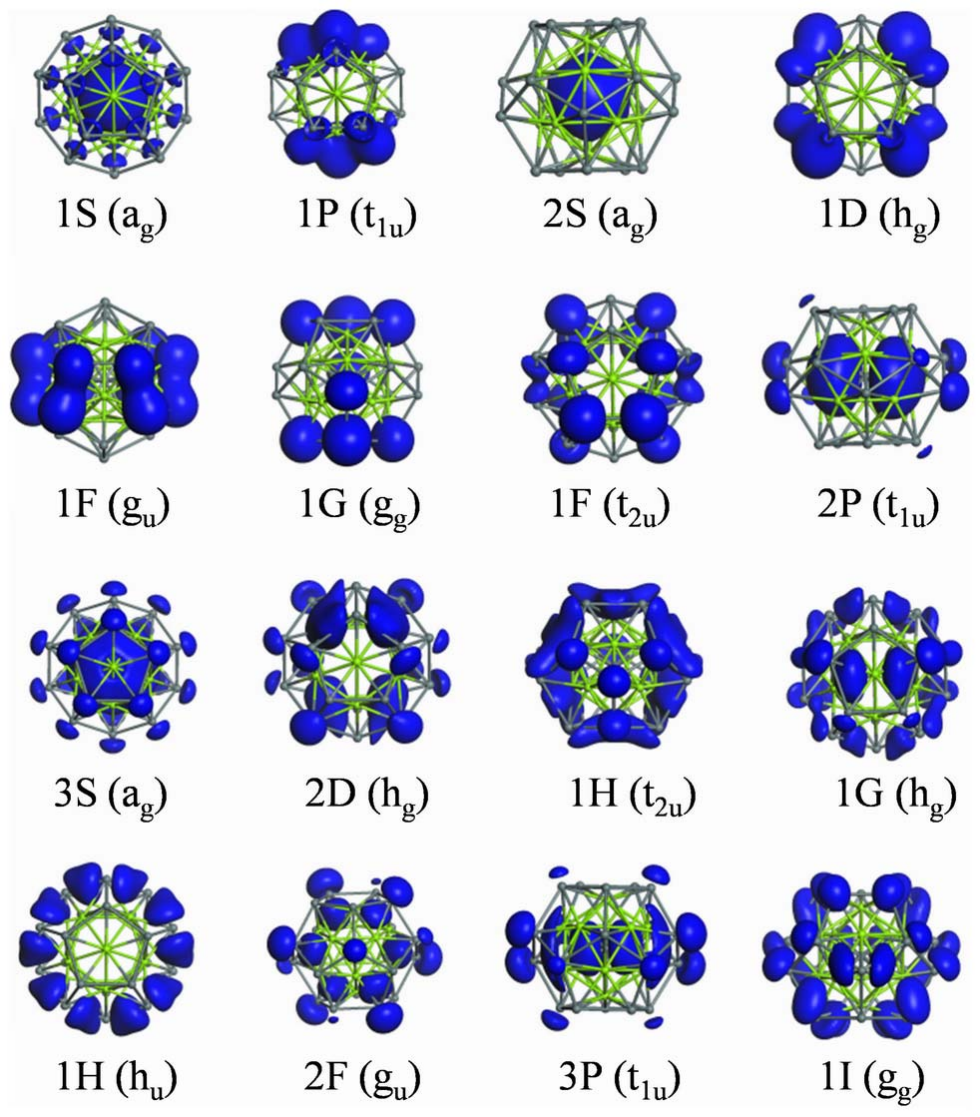
Isosurfaces for the spatial distribution of the occupied superatom orbitals of the Sn@Mg_12_@Sn_20_ cluster.

**Figure 4:**
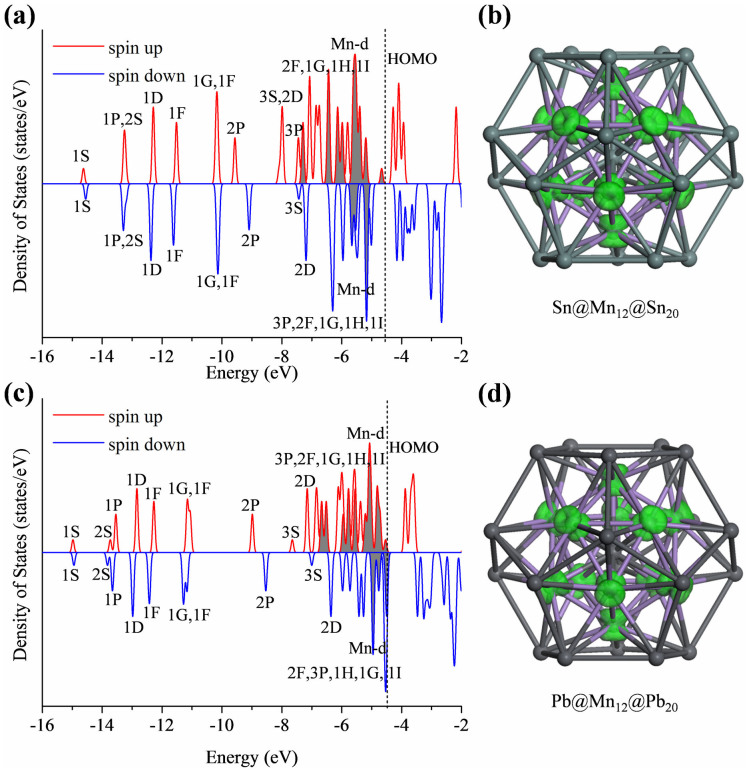
Spin-polarized density of states for (a) Sn@Mn_12_@Sn_20_ and (c) Pb@Mn_12_@Pb_20_ clusters.The occupied superatom orbitals are labelled. The d states from Mn atoms are highlighted by shadow peaks. The HOMO levels are shown by dashed lines. Atomic structure along with isosurface of spin density distribution (green areas) of (b) Sn@Mn_12_@Sn_20_ and (d) Pb@Mn_12_@Pb_20_ clusters are also presented (color code: dark gray for Sn and Pb, light purple for Mn).

**Figure 5:**
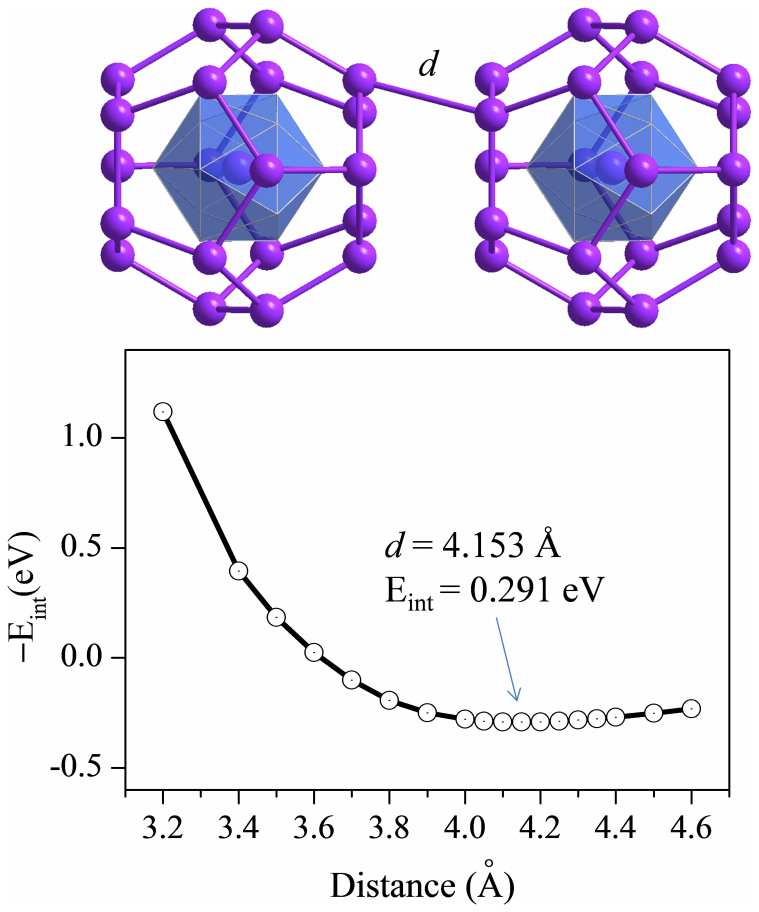
Atomic structure (upper) and binding curve (lower) for [Pb@Mn_12_@Pb_20_]_2_ dimer. The equilibrium distance (*d*) and optimal inter-cluster binding energy (E_int_) are labelled.

To confirm that the three-shell icosahedral configuration in Figure 1 is the ground state for the binary A_21_B_12_ clusters (A = Sn, Pb; B = Mg, Zn, Cd, Mn), we performed an unbiased global minimum search of two representative clusters (Sn_21_Mg_12_ and Sn_21_Zn_12_) using a genetic algorithm (GA) incorporated with DFT calculations implemented in the DMol^3^ package, which was described in our previous publication^61^. In the GA search, sixteen initial configurations were generated from scratch. Any two individuals in this population were then chosen as parents to produce a child cluster via a “cut and splice” crossover operation, followed by an optional mutation operation (either a small random displacement on each atom or exchange of a pair of different types of atoms) of 35% probability. The diversity of the populations was filtered by the inertia of each cluster. After about 3000 GA iterations, the icosahedral matryoshka structure was obtained for both Sn@Mg_12_@Sn_20_ and Sn@Zn_12_@Sn_20_ clusters as the ground state. In addition, some metastable isomers (Supplementary Figure S2) are found from GA-DFT search, but are less energetically favorable and will not be further discussed.

## Author Contributions

J.Z. designed the models and calculations. X.H., J.Z. and Y.S. did the calculations. X.H. and J.Z. prepared all the figures. J.Z., Z.C. and R.B.K. wrote the manuscript. All the authors discussed the results and commented on the manuscript.

## Supplementary Material

Supplementary Information Supplementary information

## Figures and Tables

**Table 1:** Binding energies (E_b_), formation energy (E_f_), HOMO-LUMO gaps (E_HL_), optical absorption gaps (E_og_), and the lowest allowed transition orbitals for the icosahedral matryoshka clusters A@B_12_@A_20_ (A = Sn, Pb; B = Mg, Zn, Cd)

Cluster	E_b_ (eV/atom)	E_f_ (eV/atom)	E_HL_ (eV)	E_og_ (eV)	Lowest allowed transition obitals
Sn_21_Mg_12_	2.79	0.09	1.38	1.93	HOMO → LUMO+1
Sn_21_Zn_12_	3.43	0.16	1.47	1.94	HOMO → LUMO+1
Sn_21_Cd_12_	2.60	0.21	1.29	1.84	HOMO → LUMO+1
Pb_21_Mg_12_	2.52	0.06	1.50	1.74	HOMO−2 → LUMO
Pb_21_Zn_12_	2.97	0.18	1.54	1.83	HOMO → LUMO+1
Pb_21_Cd_12_	2.28	0.16	1.27	1.72	HOMO → LUMO+1

**Table 2:** Formation energy (E_f_), HOMO-LUMO gaps (E_HL_), total magnetic moment (M_tot_) and on-site magnetic moments (A, B, C corresponding to Figure 1) for individual Sn@Mn_12_@Sn_20_ and Pb@Mn_12_@Pb_20_ clusters as well as their dimers

Cluster	E_f_(eV/atom)	E_HL_ (eV)	M_tot_(μ_B_)	M_A_ (μ_B_)	M_B_(μ_B_)	M_C_(μ_B_)
Sn_21_Mn_12_	0.346	0.659	28	−0.293	2.880	−0.698
Pb_21_Mn_12_	0.507	0.384	28	−0.369	3.013	−0.781
[Sn_21_Mn_12_]_2_	0.341	0.032	56	−0.285	2.869	−0.716
[Pb_21_Mn_12_]_2_	0.503	0.275	56	−0.367	3.009	−0.779
