# Supplementary material for: Design of Three-shell Icosahedral Matryoshka Clusters A@B12@A20 (A = Sn, Pb; B = Mg, Zn, Cd, Mn)
Source: Sci Rep. 2014 Nov 7;4:6915. doi: 10.1038/srep06915 (PMC4223685; doi:10.1038/srep06915)
Supplement: Supplementary Information [file srep06915-s1.doc]

**Design of Three-shell Icosahedral Matryoshka Clusters A@B_12_@A_20_ (A = Sn, Pb; B = Mg, Zn, Cd, Mn)**

Xiaoming Huang ^a^, Jijun Zhao a,b[[1]](#footnote-2)*, Yan Su ^a^, Zhongfang Chen ^c^, R. Bruce King ^d^

^a^ Key Laboratory of Materials Modification by Laser, Ion and Electron Beams (Dalian University of Technology), Ministry of Education, Dalian 116024, China.

^b^ Beijing Computational Science Research Center, Beijing 100089, China.

^c^ Department of Chemistry, Institute for Functional Nanomaterials, University of Puerto Rico, San Juan, PR 00923, USA.

^d^ Department of Chemistry and Center for Computational Chemistry, University of Georgia, Athens, Georgia, USA.

**Supplementary Information**

**Table S1**. On-site Mulliken charge (Q_i_) and bond lengths for the icosahedral matryoshka clusters of C@B_12_@A_20_ (C = A= Sn, Pb; B = Mg, Zn, Cd, Mn).

| Cluster | On-site charge (e) | | | Bond length (Å) | | | |
| --- | --- | --- | --- | --- | --- | --- | --- |
|  | Q_A_ | Q_B_ | Q_C_ | A-A | A-B | B-B | B-C |
| Sn_21_Mg_12_ | ‒0.242 | 0.517 | ‒1.362 | 3.427 | 3.029 | 3.150 | 2.996 |
| Sn_21_Zn_12_ | ‒0.075 | 0.171 | ‒0.245 | 3.278 | 2.907 | 2.972 | 2.827 |
| Sn_21_Cd_12_ | ‒0.098 | 0.204 | ‒0.484 | 3.463 | 3.034 | 3.289 | 3.128 |
| Sn_21_Mn_12_ | ‒0.078 | 0.210 | ‒0.957 | 3.176 | 2.870 | 2.701 | 2.569 |
| Pb_21_Mg_12_ | ‒0.187 | 0.416 | ‒1.257 | 3.545 | 3.140 | 3.231 | 3.073 |
| Pb_21_Zn_12_ | ‒0.031 | 0.084 | ‒0.404 | 3.412 | 3.033 | 3.070 | 2.919 |
| Pb_21_Cd_12_ | ‒0.049 | 0.112 | ‒0.363 | 3.578 | 3.147 | 3.351 | 3.187 |
| Pb_21_Mn_12_ | ‒0.041 | 0.148 | ‒0.950 | 3.317 | 3.016 | 2.764 | 2.629 |

**Table S2**. Covalent radius of constituent elements for the icosahedral matryoshka clusters.

|  | Sn | Pb | Be | Mg | Ca | Zn | Cd | Mn |
| --- | --- | --- | --- | --- | --- | --- | --- | --- |
| Radius (Å) | 1.39 | 1.46 | 0.96 | 1.41 | 1.76 | 1.22 | 1.44 | 1.39 |


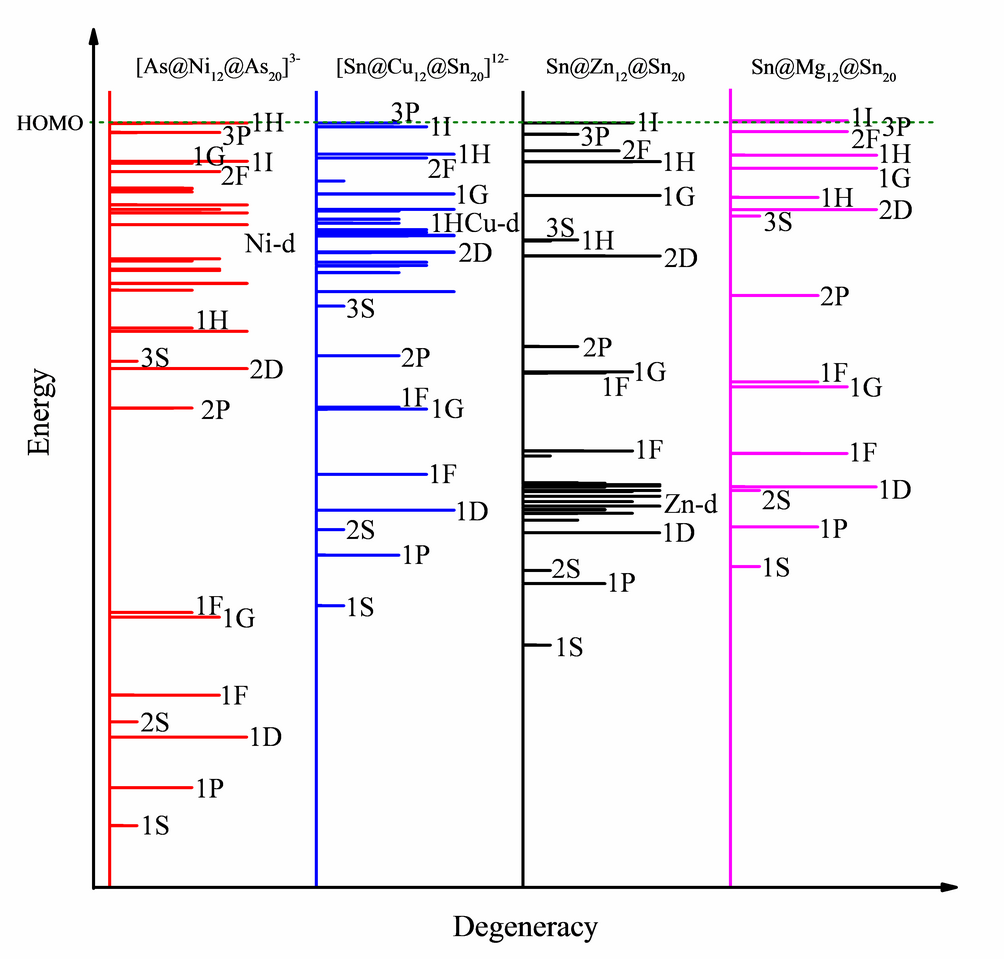


**Figure S1**. Comparison of energy levels for four kinds of icosahedral matryoshka clusters: [As@Ni_12_@As_20_]^3–^, [Sn@Cu_12_@Sn_20_]^12–^, Sn@Mg_12_@Sn_20_, and Sn@Zn_12_@Sn_20_.


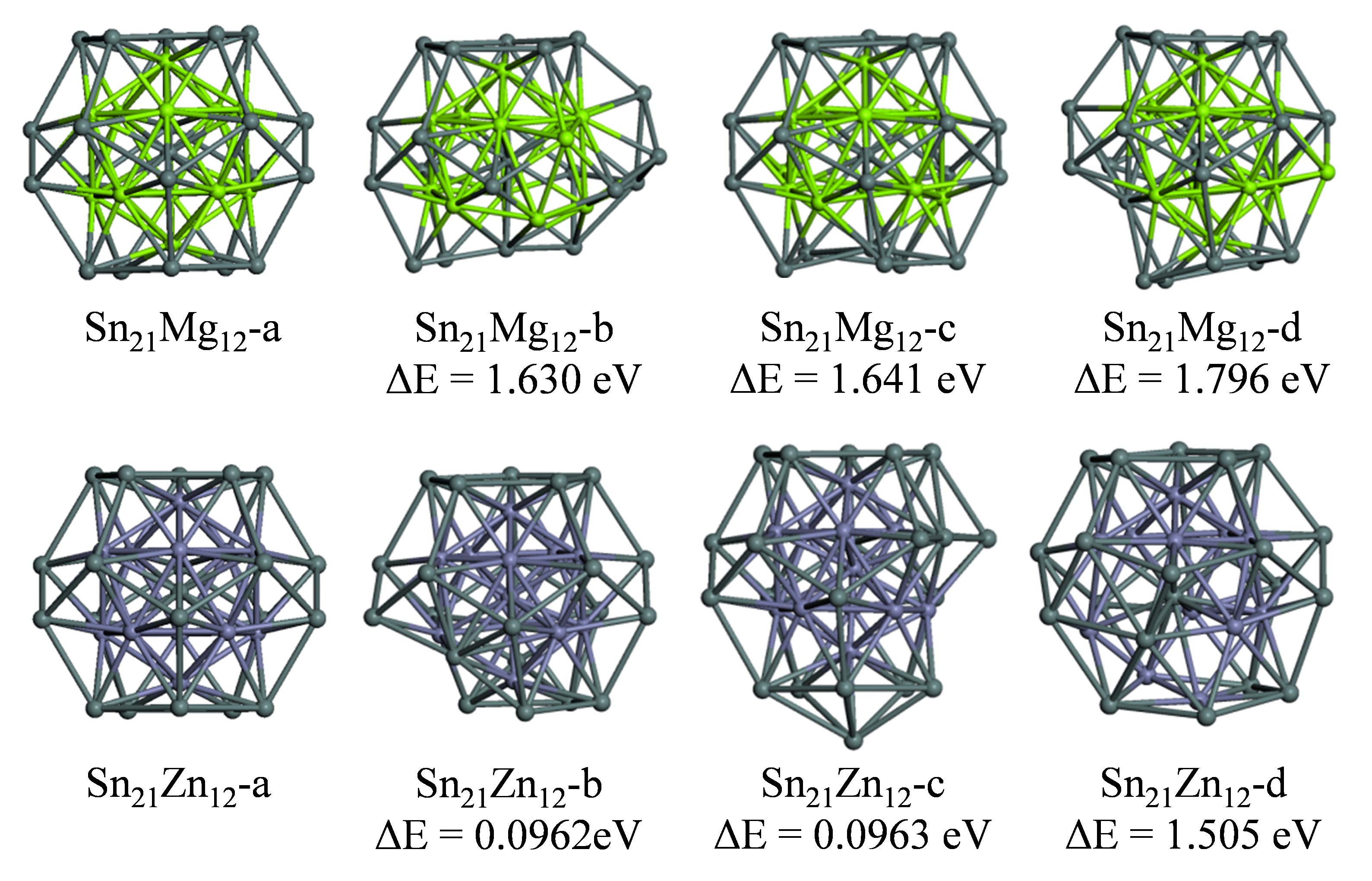


**Figure S2**. Low-lying isomer structures of Sn@Mg_12_@Sn_20_, and Sn@Zn_12_@Sn_20_ clusters. For each isomer, its energy difference to the ground state (denoted as a) is provided. Color code: dark gray for Sn, green for Mg, light purple for Zn.

1. ^^ Corresponding author: [zhaojj@dlut.edu.cn](mailto:zhaojj@dlut.edu.cn) (J. J. Zhao) [↑](#footnote-ref-2)
